# Terpenoids from *Camellia oleifera*: Structure, Biosynthesis, and Biological Activities

**DOI:** 10.3390/plants15162486

**Published:** 2026-08-16

**Authors:** Jing-Pu Tian, Bo-Lin Chen, Ji-Hong Zhang, Xiang-Nan Wang, Li Ma, Sen-Wen Deng

**Affiliations:** 1 Hunan Key Laboratory of Economic Crops Genetic Improvement and Integrated Utilization, Hunan Engineering Research Center of Lotus Deep Processing and Nutritional Health Sciences, School of Life and Health Sciences, Hunan University of Science and Technology, Xiangtan 411201, China; tianjingpu@hnust.edu.cn (J.-P.T.); jihongzh01@hnust.edu.cn (J.-H.Z.); 2 Oil-Tea Camellia Variety Creation Center, Yuelushan Laboratory, Changsha 410128, China; cbl92@njfu.edu.cn (B.-L.C.); wangxn47@163.com (X.-N.W.); 3 National Engineering Research Center of Youcha, State Key Laboratory of Woody Oil Resources Utilization, Hunan Academy of Forestry, Changsha 410004, China

**Keywords:** *Camellia oleifera*, terpenoids, triterpenoid saponins, bioactivities, biosynthesis, regulatory mechanisms

## Abstract

*Camellia oleifera* Abel., an economically significant oil-producing crop widely cultivated in southern China, is renowned for its high-quality edible oil and diverse pharmacological activities. The plant is rich in terpenoids, particularly triterpenoid saponins, which contribute to its health-promoting properties. However, a comprehensive review summarizing the diversity, biological activities, biosynthesis, and regulatory mechanisms of terpenoids in *C. oleifera* is lacking. This review systematically categorizes terpenoids identified from different parts of *C. oleifera*, elucidates their structural features, and infers their biosynthetic pathways. The biological activities of terpenoid-rich extracts and individual compounds, including antioxidant, anti-inflammatory, anticancer, antimicrobial, and hypoglycemic effects, are discussed. Furthermore, the potential applications of *C. oleifera* terpenoids in functional foods, pharmaceuticals, and agriculture are explored. This review aims to provide a valuable reference for future research and utilization of *C. oleifera* terpenoids.

## 1. Introduction

*Camellia oleifera* Abel. is a perennial shrub belonging to the family *Theaceae* and the genus *Camellia*, globally renowned as a prominent woody oil plant alongside oil palm, olive, and coconut [1]. Its seeds are the raw material for cold-pressed tea seed oil, a premium edible oil recorded in the 2015 edition of the Chinese Pharmacopoeia for its balanced unsaturated fatty acid composition and health-modulating properties [2,3]. Traditionally, all tissue parts of *C. oleifera* (seeds, defatted seed meal, roots, stems, leaves, and flower buds) have been applied in folk medicine to relieve inflammation, eliminate pathogenic toxins, and ameliorate metabolic disorders [2,4]. Beyond its nutritional and medicinal merits, this crop bears prominent economic value and broad industrial prospects. As a unique woody oil plant endemic to China, it can be cultivated on barren hills and sloped wasteland without competing with grain crops for arable land, serving as a strategic resource to secure domestic edible oil supply and reduce import dependence. *C. oleifera* represents a multi-functional woody oil crop with great potential for full resource valorization throughout its industrial chain. Nevertheless, large-scale exploitation of *C. oleifera* extracts faces multiple hidden risks, including inadequate data on chronic, reproductive and genotoxicity, restricting their food and medical applications, as well as toxic hazards to aquatic and soil organisms caused by excessive tea saponin usage [5,6].

Phytochemical studies have shown that *C. oleifera* contains a variety of chemical constituents responsible for its pharmacological effects, including triterpenoids and triterpenoid saponins, flavonoids, fatty acids, polyphenols, and polysaccharides [1]. Among these, triterpenoids and triterpenoid saponins are considered the primary bioactive constituents [5,6]. Recent studies have shown that saponins derived from *C. oleifera* exhibit significant antibacterial, anti-inflammatory, heat-clearing, and detoxifying activities, as well as notable antioxidant and anti-tumor effects [2]. In recent years, numerous studies have reported the discovery of novel triterpenoid saponins from *C. oleifera*, along with advances in elucidating their biosynthetic pathways and pharmacological activities [1]. However, statistical analyses of the literature from the past decade indicate that research on *C. oleifera* terpenoids remains relatively limited compared with studies on its polysaccharides, polyphenols, and flavonoids.

To understand the research progress and trends of the main active components of *C. oleifera*, the Web of Science database was searched using the keywords: “*Camellia oleifera*”/“triterpenoids or terpenoids”, “*Camellia oleifera*”/“polyphenols”, “*Camellia oleifera*”/“flavonoids”, and “*Camellia oleifera*”/“polysaccharides”. Statistical analysis of relevant literature published since 2015 revealed that studies on *C. oleifera* triterpenoids accounted for only 3.7% of the total publications, which was markedly lower than the proportions of polyphenols (34.0%), flavonoids (38.9%), and polysaccharides (23.4%). Luan et al. (2020) summarized 249 active compounds identified in *C. oleifera*, of which triterpenoids accounted for 53.0% [7]. Zeng et al. (2023) also verified that terpenoids are typical bioactive substances widely existing in *C. oleifera* [8]. Combined with the above research statistics, it is evident that terpenoids represented by triterpenoids are abundant in content and diverse in structural types within *C. oleifera*, whereas current academic studies pay far less attention to this class of components. Accordingly, more research attention should be devoted to the exploration and development of *C. oleifera* terpenoids.

Terpenoids (also referred to as “terpenes”) are a large class of natural products derived from isoprene units, which have attracted significant attention owing to their extensive applications in the food, pharmaceutical, cosmetic, and agricultural industries [9]. The chemistry and biochemistry of terpenoids, particularly in seed meal and tea oil, have been investigated for decades [2,6]. All terpenoids are derived from the two 5-carbon building blocks, isopentenyl diphosphate (IPP) and its enzymatically interconvertible isomer dimethylallyl diphosphate (DMAPP). These C5 precursors can be synthesized via two distinct biosynthetic pathways: the 2-C-methyl-D-erythritol-4-phosphate (MEP) pathway operating in the plastids, and the mevalonate (MVA) pathway, which is distributed across the cytoplasm, endoplasmic reticulum, and peroxisomes [10].

In general, terpenoids are classified based on the number of isoprene (C5) units they contain: monoterpenes (C10), composed of two 5-carbon building blocks, are predominantly synthesized via the MEP pathway in plastids. Diterpenes (C20), consisting of four 5-carbon building blocks, are also primarily produced through the MEP pathway. In contrast, sesquiterpenes (C15), containing three 5-carbon building blocks, are typically synthesized via the MVA pathway in the cytoplasm/endoplasmic reticulum [11]. Triterpenes (C30) are generated from six isoprene units via the MVA pathway, with squalene serving as the key precursor for subsequent cyclization reactions [9,12]. Collectively, the MEP pathway generally supplies precursors for mono- and diterpenoids in plastids, while the MVA pathway provides the foundation for sesqui- and triterpenoids in the cytosol and associated organelles [10]. More recently, genes encoding enzymes and regulators involved in terpenoid biosynthesis have been identified in *C. oleifera*, and their genomic localization, expression patterns, modification and regulatory mechanisms, and evolutionary history have been investigated [13,14,15].

Substantial progress has been made in understanding terpenoids in *C. oleifera*. Over the past decade, important advances have included the discovery of novel terpenoid structures, the elucidation of diverse mechanisms underlying their biological activities, and the extensive characterization of key genes involved in terpenoid biosynthesis. In this review, we highlight these recent advances, with particular emphasis on the structures, biosynthesis, biological activities, and potential applications of terpenoids from *C. oleifera*. Overall, this review provides a timely and comprehensive reference for the development of *C. oleifera* terpenoids as pharmaceuticals, cosmetics, and functional food ingredients, which may enhance the utilization efficiency and added value of *C. oleifera* resources, thereby promoting the sustainable development of the *C. oleifera* industry.

## 2. Types and Distribution of Terpenoids in *C. oleifera* Resources

Terpenoids have attracted considerable attention because of their extensive applications in the food, pharmaceutical, cosmetic, and agricultural industries [9,11]. All plants are capable of synthesizing a wide variety of terpenoid metabolites. To date, more than 80,000 terpenoid compounds have been identified, and many more are believed to exist [16]. Terpenoids constitute the largest class of natural compounds in living organisms, characterized by exceptional chemical and structural diversity, as well as a broad spectrum of biological functions. Their remarkable diversity arises from the extensive variability of their carbon skeletons and the wide range of biochemical modification pathways they undergo.

*C. oleifera* is distinguished not only by its production of high-quality edible oil but also for its richness in structurally diverse terpenoids. The terpenoids in *C. oleifera* are predominantly represented by triterpenoid saponins. To date, more than 70 distinct triterpenoid saponin monomers have been identified from various parts of the plant [1,5,6]. Their structural complexity and diversity primarily stem from differences in the aglycone skeletons (e.g., oleanane-type, ursane-type) and variations in the type, number, and linkage positions of sugar chains. In addition to triterpenoid saponins, the flowers of *C. oleifera* are rich in volatile terpenoids, such as monoterpenes and sesquiterpenes. These compounds contribute to the characteristic floral aroma, playing vital roles not only in attracting pollinators and facilitating reproduction but also in plant stress responses [8]. Due to the presence of abundant cytochrome P450 enzymes, acyltransferases, and glycosyltransferases in plants, oxidation, acylation, and glycosylation represent important strategies for the structural modification of terpenoids [17,18]. These modifications not only expand the diversity of terpenoid structures but also markedly enhance their water solubility and pharmacological activities [19,20].

As the center of origin and primary producer of oil-tea camellia (*C. oleifera*), China possesses abundant germplasm resources characterized by remarkable diversity and complexity in terpenoid composition. The rich species resources, such as common oil-tea camellia, Vietnamese oil-tea camellia (*C. vietnamensis* Huang/*C. drupifera* Lour.), small-fruited oil-tea camellia (*C. meiocarpa*), and Zhejiang red camellia (*C. chekiangoleosa* Hu), together with their wide geographical distribution, including unique tropical populations from Hainan Island, provide a solid foundation for the chemical diversity of terpenoids [21]. Due to differences in genetic backgrounds, species characteristics, and environmental adaptations, distinct germplasms exhibit significant variations in the types, structural modifications (e.g., glycosylation, acylation), and contents of terpenoid constituents. As a unique ecotype, the Hainan Island oil-tea camellia may harbor a special metabolic profile with distinctive terpenoid resources. For instance, previous studies have demonstrated significant differences in fatty acid profiles and antioxidant capacity among different *C. oleifera* species [22], suggesting that their secondary metabolites, particularly triterpenoid saponins, may also exhibit species-specific variation.

Various extraction techniques, including traditional maceration, ultrasound-assisted extraction, and microwave-assisted extraction, can influence the composition and structural integrity of triterpenoid saponins in crude extracts. These differences arise from variations in extraction efficiency, selectivity, and the ability to preserve thermolabile structures, thereby affecting the quality of subsequent separation and purification procedures. During purification and structural characterization, technical parameters such as chromatographic modes and HPLC conditions determine the effective separation of structurally similar saponin monomers. In addition, the resolution of two-dimensional NMR and other spectroscopic techniques directly affects the accuracy of fine structural characterization, particularly with respect to glycosidic linkage positions and acyl group configurations [6]. Therefore, methodological discrepancies in extraction and purification among studies not only contribute to variations in the types and quantities of triterpenoid saponin monomers identified but also reduce the inter-study comparability of characterized compounds.

Furthermore, the content and composition of terpenoids vary among different tissues and developmental stages of *C. oleifera*; however, systematic studies in this area remain limited. Likewise, studies focusing on volatile terpenoids, such as monoterpenes and sesquiterpenes, are still relatively scarce [8,23]. Although different triterpenoid saponins exhibit distinct biological activities, current research has largely focused on their isolation, purification, and structural characterization. As summarized in Table 1, more than 90 terpenoid compounds have been identified from *C. oleifera* to date, including monoterpenoids, sesquiterpenoids, diterpenoids, and triterpenoids (Table 1). Among these, most monoterpenoids and sesquiterpenoids are volatile compounds predominantly found in flowers. In contrast, triterpenoids-particularly triterpenoid saponins such as sasanquasaponins, camelliasaponins, theasaponins, and oleiferasaponins-are widely distributed in seeds, pomace, stems, and roots. These distinct tissue-specific accumulation patterns suggest diverse physiological functions and promising application potential of terpenoids in *C. oleifera*. Nevertheless, in-depth studies on the regulatory mechanisms underlying tissue-specific accumulation, biosynthetic pathways, and structure-activity relationships of these terpenoids remain insufficient. Furthermore, systematic comparisons of terpenoid profiles among different tissues and developmental stages are still lacking, thereby limiting the comprehensive exploitation and targeted utilization of terpenoid resources from this plant.

## 3. The Biosynthesis and Regulation of Terpenoids in *C. oleifera*

Terpenoids exhibit significant differences in distribution and accumulation among various tissues and developmental stages of *C. oleifera* [46]. Seeds, particularly at the mature stage, serve as the primary storage organs for triterpenoid saponins [1]. Flowers are rich in volatile compounds such as monoterpenes and sesquiterpenes, which contribute to their characteristic aroma and participate in pollination and defense responses [8,23]. In vegetative organs, including leaves, roots, and stems, terpenoids often function as stress-related secondary metabolites [1,47]. Their content and composition change dynamically throughout plant development. Terpenoid accumulation in *C. oleifera* flowers exhibits a characteristic pattern of an initial increase followed by a subsequent decrease, which is associated with the upregulation or activation of key biosynthetic genes during the late developmental stage [47,48]. Unlike its congener *Camellia sinensis*, systematic research on the terpenoid profiles of *C. oleifera* covering various tissues and developmental phases is still scarce, and the corresponding regulatory mechanisms remain to be further elucidated [49].

### 3.1. Triterpenoid Saponins and Volatile Terpenoids Biosynthetic Pathway

**Figure 1:**
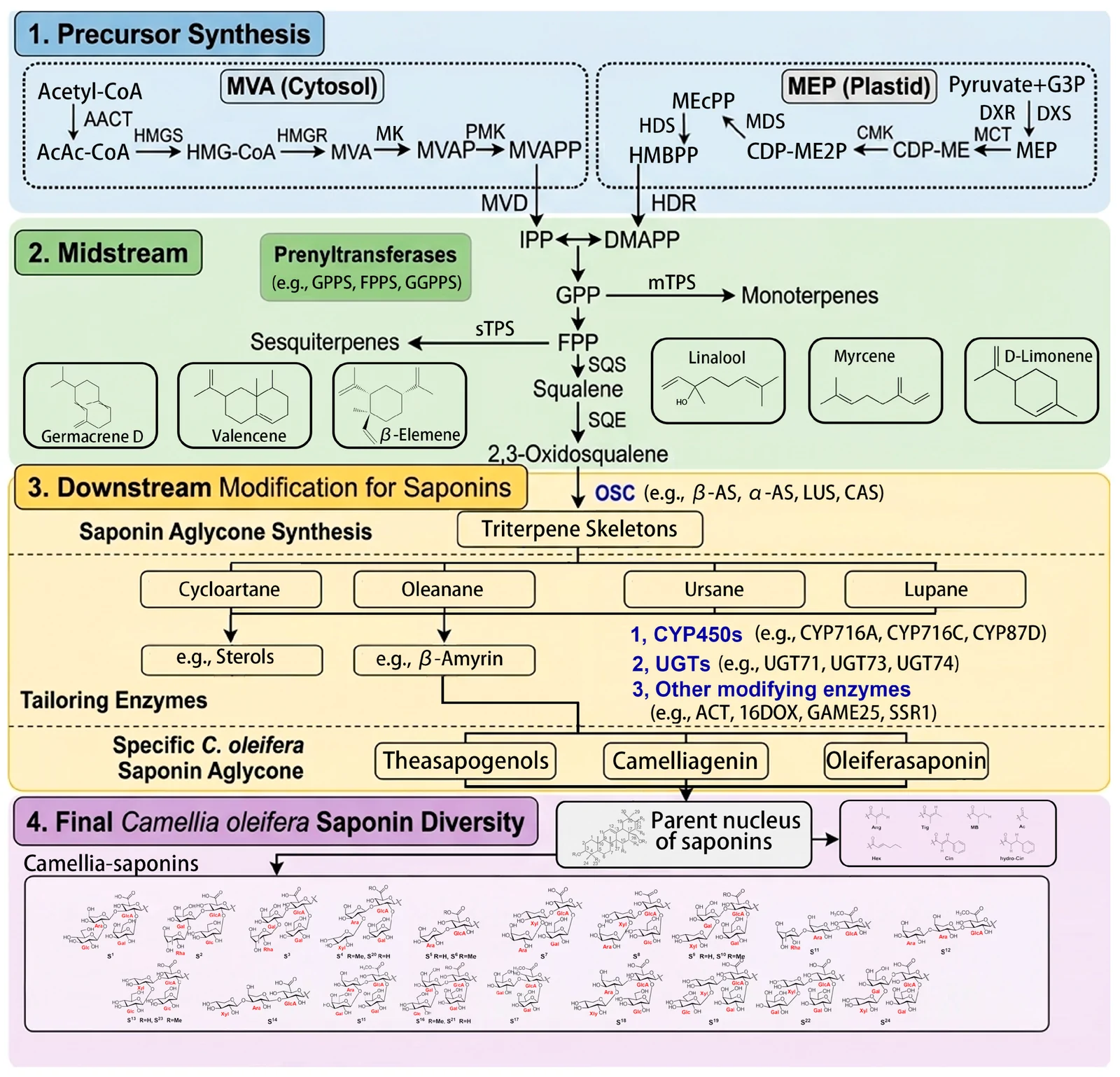
Terpenoid compound biosynthesis pathway of *C. oleifera* Abel. (Terpenoid biosynthesis in plants via mevalonate (MVA) andmethylerythritol phosphate (MEP)pathway, which generates 5-carbon terpenoid precursors, isopentenyl diphosphate and dimethylallyl diphosphate).

To date, most upstream steps of the terpenoid biosynthetic pathway have been well characterized in model plants, cereal crops, and medicinal plants [50,51]. At the molecular level, terpenoid biosynthesis is initiated via the cytoplasmic mevalonate (MVA) pathway and plastidic methylerythritol phosphate (MEP) pathway, which supply precursors for sesquiterpenoids/triterpenoids and monoterpenoids synthesis respectively [10]. The key rate-limiting steps governing C5 precursor formation are catalyzed by 3-hydroxy-3-methylglutaryl coenzyme A reductase (HMGR) and 1-deoxy-D-xylulose-5-phosphate synthase (DXS). IPP and DMAPP serve as basic C5 building blocks. They are condensed to form farnesyl pyrophosphate (FPP), which is then converted into squalene under the catalysis of squalene synthase (SQS). Squalene is further oxidized by squalene epoxidase (SQE) to generate 2,3-oxidosqualene. This compound undergoes cyclization mediated by oxidosqualene cyclases (OSCs), including β-amyrin synthase (Coβ-AS), yielding various triterpene skeletons in *C. oleifera* (Figure 1). These skeletons experience multiple complex modifications such as oxidation, acylation and glycosylation, carried out by enzyme families consisting of cytochrome P450 monooxygenases (CYP450s), acyltransferases and UDP-glycosyltransferases (UGTs) [52,53,54,55]. Meanwhile, linear precursors FPP and geranylgeranyl pyrophosphate (GGPP) are cyclized by terpene synthases (TPSs) to produce volatile sesquiterpenoids and monoterpenoids ([56,57]; Figure 1).

The biosynthesis of diverse triterpenoid saponins in *C. oleifera* is generally divided into four key stages: (1) precursor synthesis via MVA/MEP pathways to produce 2,3-oxidosqualene; (2) cyclization of 2,3-oxidosqualene catalyzed by different OSC isoforms to form various sapogenin skeletons; (3) oxidative modification of triterpene backbones by CYP450s; (4) glycosylation modification by UGTs to generate mature saponins. The first two stages refer to triterpene skeleton formation, starting from the MVA pathway and ending with the generation of 2,3-oxidosqualene that constitutes the fundamental triterpene framework. The other stages contribute to structural diversification. In general, the biosynthetic pathway of triterpenoid saponins starts with the universal precursor 2,3-oxidosqualene and yields nine subtypes of sapogenins (including theasapogenol A, theasapogenol B, theasapogenol C, theasapogenol E, theasapogenol F, camelliagenin A, camelliagenin B, oleiferasaponin B1 sapogenin, oleiferasaponin B2 sapogenin). In this process, 2,3-oxidosqualene is cyclized into various triterpene skeletons under the action of OSCs. The key branching step is mediated by the OSC gene family member β-amyrin synthase (bAS), which generates β-amyrin, which serves as the precursor of oleanane-type triterpenoid saponins that are ubiquitous pentacyclic triterpenoids in the plant kingdom and all isolated triterpenoid saponins from *C. oleifera* belong to this type. The generated skeletons are oxidized by CYP450 enzymes to form sapogenins, and further modified via UDP-glycosyltransferase (UGT)-catalyzed glycosylation. Binding with different sugar chains eventually forms abundant triterpenoid saponins in *C. oleifera* ([13,58]; Figure 1). The downstream triterpenoid saponin diversity region of Figure 1 displays three core aglycone backbones derived from β-amyrin, including Theasapogenols, Camelliagenin, and the characteristic Oleifera-type saponins (Oleiferasaponin series). The representative compounds and their corresponding molecular formulas isolated from various tissues of *C. oleifera* are listed above according to their sapogenin classification. All terpenoid products can undergo further structural modification, which substantially enriches the chemical diversity of *C. oleifera* terpenoids, especially triterpenoid saponins, and elevates their water solubility and biological activities [19,20]. 

### 3.2. The Regulation of Terpenoid Metabolism in C. oleifera

In plants, the diversity of terpenoid compounds arises not only from their intrinsically diverse structures, substrates, enzymatic reaction types, and sites of biosynthesis, but also from the exceptionally complex and multilayered regulatory networks controlling gene expression. Beyond the inherent complexity of terpenoid biosynthetic pathways, terpenoid metabolism is precisely regulated by transcription factors, including members of the MYB (v-myb avian myeloblastosis viral oncogene homolog), bHLH (basic helix-loop-helix) and WRKY families. These transcription factors can activate or repress the expression of key biosynthetic genes, such as *OSCs*, *TPSs*, *HMGR*, and *DXS*, often through the formation of regulatory complexes [11,59]. At the same time, environmental factors, including light, temperature, and biotic stresses caused by pests and pathogens, can significantly influence the activity of these regulators, thereby reshaping terpenoid biosynthetic profiles. Recent studies have further revealed that epigenetic regulation, including DNA methylation and histone modification, plays a fundamental role in determining the spatiotemporal specificity of terpenoid biosynthetic gene expression [60,61]. Together, these interconnected regulatory layers constitute a dynamic and plastic network that enables plants to produce a vast and adaptable repertoire of terpenoid compounds, finely tuned to developmental requirements and environmental changes.

Terpenoid biosynthesis in *C. oleifera* also exhibits pronounced tissue specificity and spatiotemporal regulation. One of the most representative advances has been the elucidation of terpenoid metabolism during floral organ development. Studies have shown that, during the late stage of flower bud development, monoterpene biosynthesis is specifically enriched in pistils and stamens [8]. Dynamic transcriptomic analyses further revealed that the expression of genes encoding DXS and multiple TPSs exhibits distinct temporal fluctuations. The expression peaks of these genes closely coincide with floral organ maturation and pollination readiness, suggesting that monoterpene volatiles play an important role in attracting pollinators. Such precise temporal regulation ensures the efficient allocation of metabolic resources and the optimization of ecological functions [48].

Environmental factors also serve as important external signals that reshape terpenoid metabolic networks in *C. oleifera*. Under abiotic stress conditions, alkali stress can significantly activate sesquiterpenoid and triterpenoid biosynthetic pathways in *C. oleifera* leaves. The upregulation of related biosynthetic genes likely represents a physiological defense and adaptive response mechanism [62]. Under biotic stress conditions, pest infestation combined with high-temperature stress induces significant changes in the levels of defense-related secondary metabolites, including terpenoids, in *C. oleifera* seeds. These findings demonstrate that terpenoid biosynthesis, as an important component of plant stress resistance, is precisely regulated through complex transcriptional reprogramming networks triggered by stress signals. Recent multi-omics studies have further revealed that the biosynthetic networks of oils (triacylglycerols and fatty acids) and terpenoids, along with other secondary metabolites, in *C. oleifera* kernels are closely interconnected and coordinately regulated by the availability of upstream carbon sources [63].

Although co-expression network analysis has preliminarily identified eight transcription factors potentially involved in theasaponin biosynthesis in *C. oleifera*, their specific identities and functions remain largely uncharacterized. Based on the general regulatory principles of plant specialized metabolism and findings from closely related species, transcription factors belonging to the MYB, bHLH and WRKY families are considered potential key regulators [59,60]. However, the precise regulatory relationships and molecular mechanisms between these candidate transcription factors and downstream key biosynthetic enzymes, such as CoHMGR, Coβ-AS, CYP450s, and UGTs, still require systematic validation through genetic and molecular biological approaches. In the future, functional characterization of these transcription factors and elucidation of their regulatory networks will be crucial for comprehensively understanding the transcriptional regulatory mechanisms underlying theasaponin biosynthesis [13,64].

## 4. Biological Activities and Potential Applications of Terpenoids from *C. oleifera*

**Figure 2:**
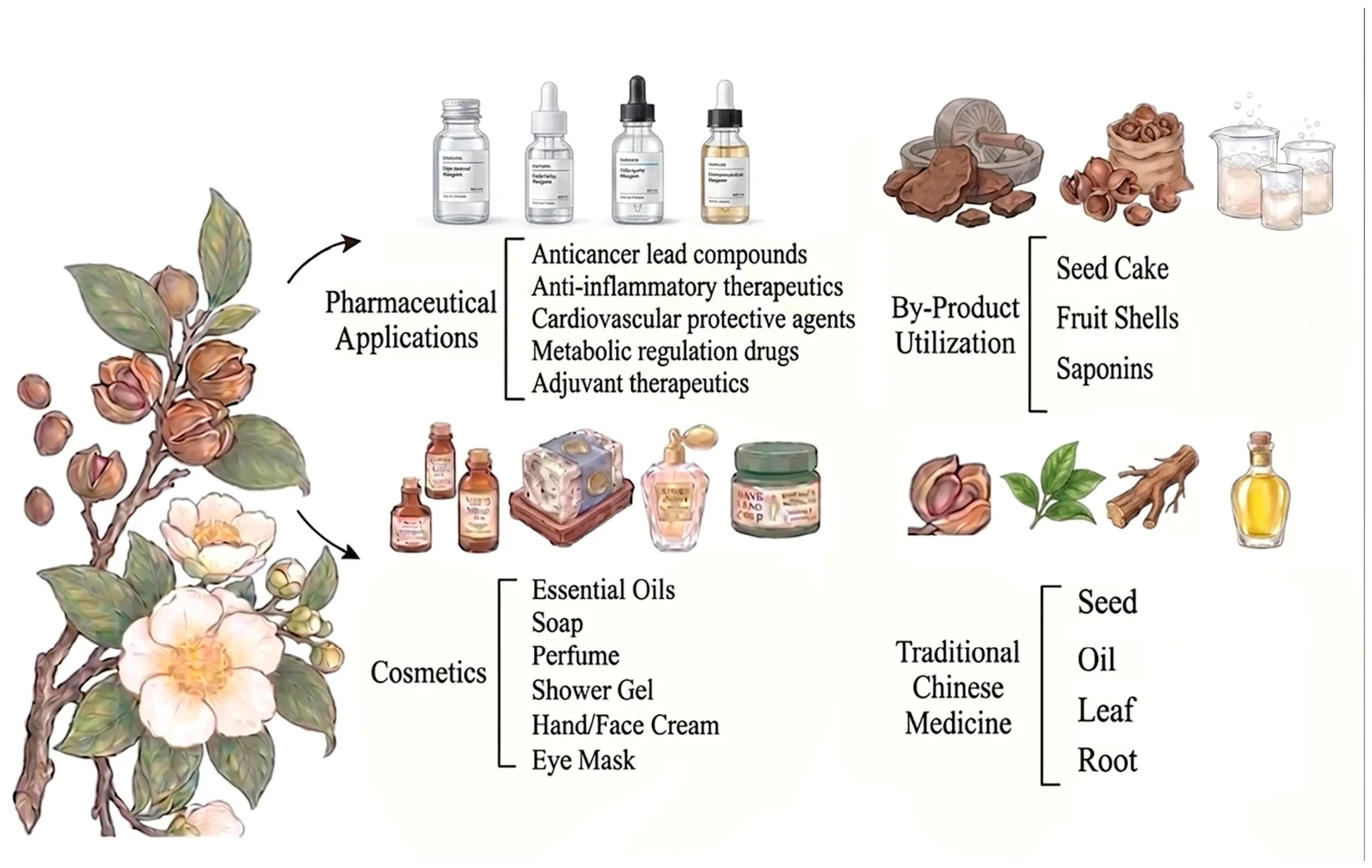
Applications of *C. oleifera*.

The tissues of *C. oleifera* are rich in structurally diverse terpenoid compounds, which can be broadly classified into oleanane-type triterpenoid saponins (theasaponins) and volatile terpenoids predominantly composed of monoterpenes and sesquiterpenes. These compounds are not only important secondary metabolites in oil-tea camellia but also represent highly promising natural resources for the pharmaceutical, agricultural, and cosmetic industries because of their broad spectrum of biological activities ([6,8]; Figure 2).

### 4.1. Biological Activities of Major Terpenoid Components

**Figure 3:**
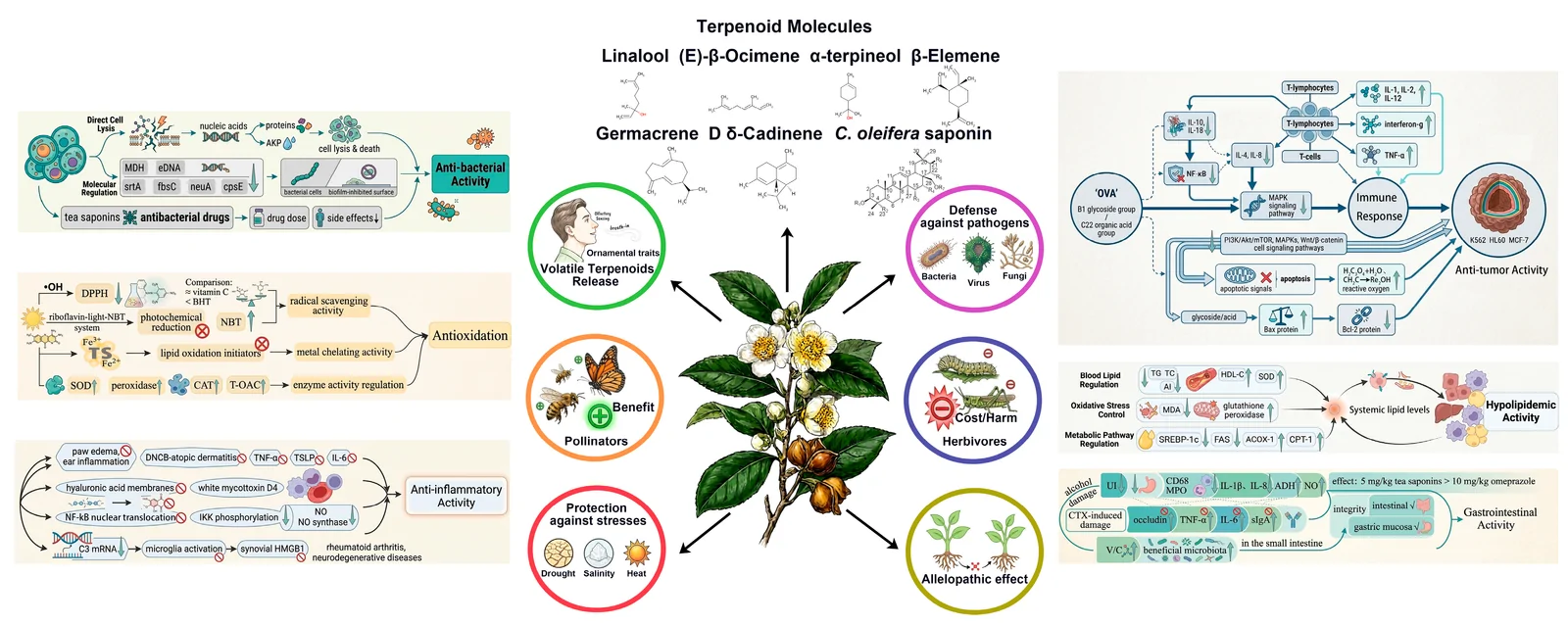
Schematic illustration of ecological roles and pharmacological bioactivities of terpenoids in *C. oleifera*.

Terpenoids from *C. oleifera* are broadly categorized into triterpenoid saponins and volatile mono- and sesquiterpenoids, both of which exhibit multifaceted bioactivities essential to the scope of this review. Current research has predominantly centered on the pharmacological properties of oleanane-type triterpenoid saponins, establishing their remarkable antitumor, anti-inflammatory, antioxidant, antibacterial, and metabolic regulatory activities ([7]; Figure 3). However, the precise molecular mechanisms underpinning these broad physiological effects, such as specific target proteins, downstream signaling cascades, and gene regulatory networks, remain fragmented across the literature and require systematic integration. 

Beyond non-volatile saponins, volatile monoterpenes and sesquiterpenoids represent another crucial yet underrepresented class of bioactives. These volatile constituents not only orchestrate defensive strategies against pathogens and herbivores but also emit floral volatiles that attract pollinators for reproductive success [11]. Moreover, their secondary bioactive roles, including antioxidant potential and ecological pest control, warrant equal mechanistic scrutiny. In this section, we provide a comprehensive breakdown of the functional profiles and underlying molecular mechanisms for both triterpenoid saponins and volatile terpenoids, while identifying key knowledge gaps in their therapeutic and ecological applications (Figure 3).

#### 4.1.1. Core Pharmacological Activities of Triterpenoid Saponins

Triterpenoid saponins represent the most extensively studied and biologically important terpenoid fraction in *C. oleifera*. They exhibit a wide range of pharmacological activities, including antitumor, anti-inflammatory, immunomodulatory, antioxidant, antimicrobial, antithrombotic, anthelmintic, and cholesterol-lowering effects. Multiple studies have demonstrated that oleanane-type triterpenoid saponins derived from *C. oleifera* can effectively inhibit the proliferation of various cancer cell types, including liver, lung, breast, and cervical cancer cells [38]. These effects are primarily associated with the induction of apoptosis and cell cycle arrest [65,66]. Saponins isolated from the fruit shells of *C. oleifera* have been shown to significantly suppress the production of inflammatory mediators such as TNF-α and IL-6 through modulation of key signaling pathways, including nuclear factor-kappa B (NF-κB) and mitogen-activated protein kinase (MAPK) pathways (p38, c-Jun N-terminal kinase (JNK), extracellular signal-regulated kinase (ERK)), thereby exhibiting pronounced anti-inflammatory effects [66,67]. In addition, saponins extracted from camellia seed cake (meal) display strong free radical scavenging activities in DPPH and ABTS assays, which may contribute to their health-promoting and restorative functions [4,6]. Previous studies have further reported that *C. oleifera* saponins possess antimicrobial, antithrombotic, anthelmintic, and cholesterol-lowering activities, highlighting their broad biological potential [33,65,66,67,68,69,70].

#### 4.1.2. Ecological and Potential Biological Activities of Volatile Terpenoids

Research on the biological activities of volatile terpenoids that contribute to the characteristic aroma of *C. oleifera* flowers and leaves is less systematic than that on saponins. While *Camellia sinensis* releases large quantities of floral volatile substances to lure pollinating insects [49], *C. oleifera* presents totally different pollination ecological traits. *C. oleifera* pollen contains high levels of toxic triterpenoid saponins that are lethal to honeybee larvae, suggesting an “anti-bee” pollination syndrome, possibly associated with bird visitation, though experimental evidence is lacking [71].

To date, no study has systematically characterized the floral volatile terpenoids of *C. oleifera* or experimentally validated the role of specific scent compounds in pollinator attraction. However, based on their chemical properties and preliminary evidence, their core functions are known to center on ecological defense. Components such as D-limonene, γ-terpinene, geraniol, β-myrcene and germacrene D have been identified in *C. oleifera* flowers. The antimicrobial and insecticidal activities of D-limonene, γ-terpinene, and geraniol have been well documented in various plants [72], suggesting that their presence in *C. oleifera* flowers may contribute to ecological defense functions [8]. Some monoterpenes and sesquiterpenes, like β-myrcene and germacrene D, have been reported in other plant systems to possess anti-inflammatory and antioxidant activities, suggesting similar potential biological activities in *C. oleifera* [72,73,74].

Volatile terpenoids constitute one of the largest and most chemically diverse classes of compounds in plant floral scents and play a fundamental role in shaping aroma profiles. In *C. oleifera*, key monoterpenes such as linalool, linalyl acetate, and (−)-carvone serve as important flavor and fragrance constituents [8]. These compounds not only contribute to the characteristic floral scent of *C. oleifera* but also enhance its sensory quality, thereby increasing the economic value of the plant and its derived products.

### 4.2. Exploitation Pathways and Future Prospects of C. oleifera Terpenoids

The diverse biological activities of terpenoids from *C. oleifera* position them as valuable resources with broad application potential in pharmaceutical, agricultural, cosmetic, and industrial sectors ([7], Figure 2).

#### 4.2.1. Pharmaceutical Applications and Prospective Development

Triterpenoid saponins, the most extensively studied terpenoid fraction in *C. oleifera*, exhibit a broad spectrum of pharmacological activities that support their development as therapeutic agents and bioactive pharmaceutical ingredients [1,7]. The significant antitumor, anti-inflammatory, and antioxidant properties of these compounds have been well documented [1,75,76]. Mechanistically, oleanane-type triterpenoid saponins exert cytotoxic effects against various cancer cell lines through the induction of apoptosis and cell cycle arrest. Structure-activity relationship (SAR) studies have revealed that substitution sites at the C-3, C-15, C-16, C-21, C-22, C-23, and C-28 positions of pentacyclic triterpenoids are critical for their antitumor activity [1]. Furthermore, these saponins exhibit cardiovascular protective effects, neuroprotective effects, as well as hypoglycemic and hypolipidemic activities [7,75]. Their anti-inflammatory effects are primarily mediated through modulation of key signaling pathways, including NF-κB and MAPK, leading to reduced production of pro-inflammatory cytokines such as TNF-α and IL-6.

Notably, most triterpenoid saponins possess hemolytic activity, which restricts their direct use as functional food or dietary additives. Instead, their strong and diverse bioactivities render *C. oleifera* triterpenoids promising candidates for novel pharmaceutical lead compounds, targeted drug delivery systems, or adjuvant therapeutic agents. They hold substantial potential for application in cancer adjuvant therapy, chronic inflammatory diseases, cardiovascular protection, and related healthcare fields ([75]; Figure 2).

#### 4.2.2. Green Agriculture Applications

The natural surfactant properties of tea saponins, combined with their antimicrobial, insecticidal, and nematicidal activities, position them as eco-friendly alternatives to synthetic agrochemicals in sustainable agriculture [7]. Tea saponins exhibit potent insecticidal effects against soft-bodied pests, including nematodes, snails, and certain insects, by disrupting pest cell membranes while remaining non-toxic to beneficial organisms such as bees and ladybirds. They have been effectively applied to control soil-borne diseases caused by pathogens such as *Fusarium* and *Rhizoctonia* [76]. In addition, tea saponins exhibit allelopathic effects that inhibit weed growth, suggesting their potential for organic weed management as alternatives to chemical herbicides [77,78].

Beyond pest control, tea saponins can improve soil health by promoting microbial diversity, enhancing soil aeration, and increasing nutrient availability through their surfactant properties [79,80]. Their biodegradability and environmental safety are consistent with integrated pest management (IPM) strategies and global organic agriculture standards. Moreover, the ability of tea saponins to remove pesticide residues from the surfaces of fruits and vegetables further expands their potential applications in post-harvest management and food safety [81].

#### 4.2.3. Daily Chemical and Cosmetic Applications

The amphiphilic molecular structure of tea saponins, comprising hydrophilic sugar moieties and hydrophobic aglycone groups, confers excellent surface-active properties, including detergency, foaming, emulsification, and wetting capabilities [1,7]. These properties, combined with their natural origin and biodegradability, make tea saponins valuable ingredients in the development of eco-friendly cleaning products, cosmetics, and personal care formulations [80]. The mild cleansing and emulsifying properties of saponins, together with the natural fragrance contributed by volatile terpenoids such as linalool, linalyl acetate, and other aromatic compounds, can be utilized to develop high-end skincare products, natural perfumes, and sustainable personal care products [1,8]. Furthermore, the antioxidant properties of tea saponins provide additional benefits in cosmetic formulations by protecting skin against oxidative damage.

Notably, *C. oleifera* also represents a promising source of high-value essential oils and seed oil for advanced cosmetic applications. The flower-derived essential oil, rich in monoterpenes and sesquiterpenes [8], exhibits a delicate, long-lasting floral aroma and notable antioxidant, anti-inflammatory, and skin-soothing properties, making it highly suitable for natural perfumes, aromatic skincare, and scalp care products. Collectively, tea saponins and flower essential oils form a versatile natural ingredient system, offering great potential for creating green, mild, and multifunctional daily chemicals and cosmetic products with high added value.

#### 4.2.4. Comprehensive Resource Utilization and Industrial Applications

The extraction of oil from *C. oleifera* seeds generates substantial quantities of by-products, including seed cake and fruit shells, both of which contain high levels of bioactive terpenoids. The content of tea saponins in seed cake ranges from 10% to 20%, representing a valuable resource for valorization [75,78]. The development of efficient and environmentally friendly extraction technologies, including aqueous enzymatic extraction, ultrasound-assisted extraction, microwave-assisted extraction, and supercritical fluid extraction, has improved the recovery of these bioactive compounds while preserving their functional properties [82].

Due to the hemolytic toxicity of seed cake-derived saponins to fish, defatted camellia seed cake has long been used as a selective piscicide for pond treatment in shrimp and crab aquaculture [83]. Beyond conventional uses, tea saponins also show promising prospects in environmental management, such as degrading pollutants in contaminated soils and eliminating pesticide residues on agricultural products. The full utilization of processing by-products not only boosts resource efficiency but also elevates the overall economic value of the *C. oleifera* industry chain, in line with circular economy principles and sustainable development goals [76,80].

In conclusion, the diverse biological activities and physicochemical properties of *C. oleifera* terpenoids, particularly triterpenoid saponins, provide extensive opportunities for high-value applications across multiple industries. Future research should focus on elucidating structure-activity relationships, conducting clinical validations, optimizing extraction technologies, and developing scalable industrial processes to fully realize the potential of these natural bioactive compounds [1,6,80].

## 5. Conclusions and Prospects

*Camellia oleifera*, a woody oil crop with high economic and application value, has attracted increasing attention in recent years due to the rich diversity of terpenoids it produces. This review systematically summarizes current research on terpenoids from *C. oleifera*, with a focus on oleanane-type triterpenoid saponins and volatile terpenoids primarily composed of monoterpenes and sesquiterpenes. To date, more than 70 distinct triterpenoid saponin monomers, along with numerous volatile constituents, have been identified from various tissues of *C. oleifera* [1]. Their structural diversity arises mainly from variations in the aglycone skeleton and subsequent modifications, including oxidation, acylation, and glycosylation. The biosynthesis of these terpenoids primarily follows the classical MVA and MEP pathways, which are precisely regulated by a multi-layered, complex network involving transcription factors, environmental signals, and epigenetic mechanisms.

In terms of biological activities, triterpenoid saponins have been confirmed to possess a range of pharmacological effects, including antitumor, anti-inflammatory, antioxidant, and antimicrobial activities [77]. Meanwhile, volatile terpenoids play important roles in ecological defense and pollinator attraction. Based on these properties, terpenoids from *C. oleifera* exhibit broad application prospects in pharmaceuticals, functional foods, green agriculture, and daily chemical industries. Although considerable progress has been achieved, significant gaps and challenges remain in this field. Future research should focus on the following key directions for further breakthroughs.

### 5.1. Deepening Fundamental Research and Deciphering Regulatory Networks

Current understanding of the tissue-specific and developmentally dynamic accumulation patterns of *C. oleifera* terpenoids, particularly volatile constituents, remains fragmented [8,13]. Future studies should employ integrated multi-omics approaches, including transcriptomics, metabolomics, and proteomics, to construct comprehensive metabolic profiles across different tissues and developmental stages, as well as under various stress conditions. For example, alkali stress has been shown to activate sesquiterpenoid and triterpenoid biosynthetic pathways [62].

Furthermore, although preliminary screening has identified candidate regulatory components, including 11 CYP450s, 14 UGTs, and eight transcription factors potentially involved in theasaponin biosynthesis [13], the specific functions of key transcription factor families such as MYB, bHLH, and WRKY, as well as their downstream target genes (e.g., CoHMGR, Coβ-AS, CYP450s, and UGTs), remain unclear. These transcription factors act as core regulatory nodes that integrate phytohormone signals and environmental cues to modulate gene expression and form sophisticated regulatory networks. To fully decipher the underlying mechanisms, multiple molecular approaches are urgently required for functional verification. Traditional molecular techniques including yeast one-hybrid (Y1H), dual-luciferase reporter assay and chromatin immunoprecipitation (ChIP) can verify the direct binding between transcription factors and promoter sequences of downstream structural genes. Gene manipulation tools such as CRISPR-Cas9 gene editing, gene overexpression and RNA interference are applied to characterize in vivo gene functions. In addition, yeast two-hybrid (Y2H), bimolecular fluorescence complementation (BiFC) and co-immunoprecipitation (Co-IP) are utilized to explore protein–protein interactions within hormone and stress signaling pathways. Combined with exogenous phytohormone treatment and transcriptome analysis, these methods collectively clarify how transcription factors mediate signal transduction and regulate the biosynthesis of terpenoids. In the future, the integration of the above approaches with multi-omics technologies will further unravel the sophisticated regulatory networks underlying terpenoid metabolism in *C. oleifera*.

### 5.2. Integrating Synthetic Biology with Germplasm Resource Utilization

Building upon the elucidation of key biosynthetic enzyme functions and regulatory mechanisms, synthetic biology strategies can be employed to reconstruct and optimize terpenoid biosynthetic pathways in microbial or plant chassis systems [84]. This approach provides a promising strategy for the green and sustainable large-scale production of these high-value compounds [85]. In addition, systematic evaluation of terpenoid components across different germplasm resources, particularly unique local varieties from regions such as Hainan, Guangxi, and Hunan, should be strengthened. The mining of superior alleles, combined with molecular marker-assisted breeding techniques, may facilitate the development of new cultivars with enhanced accumulation of bioactive compounds and improved stress resistance [61,84,85].

### 5.3. Strengthening Structure-Activity Relationship Studies and Industrial Application Research

Current research on the bioactivities of *C. oleifera* triterpenoid saponins largely relies on crude extracts or mixtures. Future studies should prioritize the evaluation of in vitro and in vivo efficacy of single, pure compounds and delve deeper into their structure-activity relationships (SAR) to identify key active functional groups. Notably, substitution sites at the C-3, C-15, C-16, C-21, C-22, C-23, and C-28 positions of pentacyclic triterpenoids have been identified as critical for antitumor activity, providing a foundation for rational drug design and structural optimization [1]. Concurrently, application-oriented research and development should be intensified to explore the utilization of these active constituents in novel biopesticides, animal feed additives, and functional skincare products. Furthermore, the establishment of integrated, efficient, and environmentally friendly extraction processes for bioactive compounds from processing by-products-including camellia seed cake (containing 10–20% saponins), fruit shells, and leaves-is essential for further enhancing the added value of the *C. oleifera* industry [76].

In conclusion, terpenoids from *C. oleifera* represent a reservoir of significant scientific value and application potential. Through interdisciplinary collaboration and technological innovation-integrating advances in genomics, metabolomics, synthetic biology, and green chemistry-the systematic elucidation of their biosynthetic and regulatory mechanisms, as well as the promotion of their high-value utilization, will not only provide new momentum for related industries but also serve as an important model for research in plant specialized metabolism. Apart from pathway engineering and green extraction, targeted techniques for stabilizing, retaining and quantitatively controlling terpenoids deserve more attention: optimized harvesting timing, low-temperature drying, mild separation processes and low-temperature sealed storage effectively avoid the degradation of active terpenoids during processing and preservation, while controlled fermentation and molecular modification can precisely regulate the yield of target terpenoid compounds. Comprehensive mastery of such preservation and quantitative regulation strategies lays a solid foundation for standardized industrial production of tea-derived pharmaceuticals and cosmetics. Continued exploration of this remarkable natural resource is expected to yield both fundamental scientific insights and practical applications beneficial to human health and sustainable agriculture [1,80].

## Figures and Tables

**Table 1:** Chemical structures of terpenoids from *C. oleifera*.

Number	Name	Molecular Formula	Part	Reference
Monoterpenoids				
1	D-Limonene	C10H16	flowers	Gan et al., 2013 [23]; Zeng et al., 2023 [8]
2	Myrcene	C10H16	flowers	Gan et al., 2013 [23]; Zeng et al., 2023 [8]
3	(*Z*)-Ocimene	C10H16	flowers	Gan et al., 2013 [23]
4	(*E*)-*β*-Ocimene	C10H16	flowers	Gan et al., 2013 [23]
5	Linalool	C10H18O	flowers	Gan et al., 2013 [23]
6	*Trans*-anethole	C10H12O	flowers	Gan et al., 2013 [23]
7	hotrienol	C10H16O	flowers	Zeng et al., 2023 [8]
8	*α*-terpineol	C10H18O	flowers	Zeng et al., 2023 [8]
9	lilac alcohol	C10H18O2	flowers	Zeng et al., 2023 [8]
10	7-Octen-4-ol, 2-methyl-6-methylene-	C10H18O	flowers	Zeng et al., 2023 [8]
11	(2S,4R)-4-Methyl-2-(2-methylprop-1-en-1-yl)tetrahydro-2H-pyran	C10H18O	flowers	Zeng et al., 2023 [8]
12	Furan, 3-(4-methyl-3-pentenyl)-	C10H14O	flowers	Zeng et al., 2023 [8]
13	(-)-Carvone	C10H14O	flowers	Zeng et al., 2023 [8]
14	Cyclohexanone, 5-methyl-2-(1-methylethyl)-	C10H18O	flowers	Zeng et al., 2023 [8]
15	Carvenone	C10H16O	flowers	Zeng et al., 2023 [8]
16	6-Octen-1-ol, 7-methyl-3-methylene-	C10H18O	flowers	Zeng et al., 2023 [8]
17	endo-Borneol	C10H18O	flowers	Zeng et al., 2023 [8]
18	Thujone	C10H16O	flowers	Zeng et al., 2023 [8]
19	*γ*-Terpinene	C10H16	flowers	Zeng et al., 2023 [8]
20	2,6-Octadienal, 3,7-dimethyl-, (*Z*)-	C10H16O	flowers	Zeng et al., 2023 [8]
21	Cyclohexene, 1-methyl-4-(1-methylethylidene)-	C10H16	flowers	Zeng et al., 2023 [8]
22	7-Octen-4-ol, 2-methyl-6-methylene-, (*S*)-	C10H18O	flowers	Zeng et al., 2023 [8]
23	6-Octen-1-ol, 3,7-dimethyl-, formate	C11H20O2	flowers	Zeng et al., 2023 [8]
24	Cyclohexanol, 1-methyl-4-(1-methylethyl)-, *trans*-	C10H20O	flowers	Zeng et al., 2023 [8]
25	1-Cyclohexene-1-carboxylic acid, 4-(1-methylethenyl)-	C10H14O2	flowers	Zeng et al., 2023 [8]
26	Isopinocarveol	C10H16O	flowers	Zeng et al., 2023 [8]
27	Pinocarvone	C10H14O	flowers	Zeng et al., 2023 [8]
28	Bicyclo[3.1.0]hexan-2-ol, 2-methyl-5-(1-methylethyl)-, (1.α.,2.α.,5.α.)-	C10H18O	flowers	Zeng et al., 2023 [8]
29	1,5-Heptadien-4-one, 3,3,6-trimethyl-	C10H16O	flowers	Zeng et al., 2023 [8]
30	(E)-4,8-Dimethylnona-1,3,7-triene	C11H18	flowers	Zeng et al., 2023 [8]
31	2,6-Octadienal, 3,7-dimethyl-, (E)-	C10H16O	flowers	Zeng et al., 2023 [8]
32	Geraniol	C10H18O	flowers	Zeng et al., 2023 [8]
33	Linalyl acetate	C12H20O2	flowers	Zeng et al., 2023 [8]
34	2,6-Dimethyl-2-*trans*-6-octadiene	C10H18	flowers	Zeng et al., 2023 [8]
35	Cyclohexane, 1-methylene-4-(1-methylethenyl)-	C10H16	flowers	Zeng et al., 2023 [8]
36	*β*-Phellandrene	C10H16	flowers	Zeng et al., 2023 [8]
37	2-Cyclohexen-1-one, 3-methyl-6-(1-methylethenyl)-, (*S*)-	C10H14O	flowers	Zeng et al., 2023 [8]
38	*cis*-Dihydrocarvone	C10H16O	flowers	Zeng et al., 2023 [8]
39	Bicyclo[3.1.1]hept-2-ene-2-carboxaldehyde, 6,6-dimethyl-	C10H14O	flowers	Zeng et al., 2023 [8]
40	7-Octen-2-ol, 2,6-dimethyl-	C10H20O	flowers	Zeng et al., 2023 [8]
41	1,3-Cyclohexadiene-1-carboxaldehyde, 2,6,6-trimethyl-	C10H14O	flowers	Zeng et al., 2023 [8]
42	*trans*-Carveol	C10H16O	flowers	Zeng et al., 2023 [8]
43	Verbenone	C10H14O	flowers	Zeng et al., 2023 [8]
44	1,3,8-p-Menthatriene	C10H14	flowers	Zeng et al., 2023 [8]
45	Carveol	C10H16O	flowers	Zeng et al., 2023 [8]
46	D-Verbenone	C10H14O	flowers	Zeng et al., 2023 [8]
47	2,6,6-trimethyl-1-Cyclohexene-1-carboxaldehyde	C10H16O	flowers	Zeng et al., 2023 [8]
Sesquiterpenoids				
1	*β*-Elemene	C15H24	flowers	Gan et al., 2013 [23]
2	Germacrene D	C15H24	flowers	Gan et al., 2013 [23]; Zeng et al., 2023 [8]
3	Valencene	C15H24	flowers	Gan et al., 2013 [23]
4	δ-Cadinene	C15H24	flowers	Gan et al., 2013 [23]
5	Farnesol isomer A	C15H26O	flowers	Gan et al., 2013 [23]
6	Farnesol isomer B	C15H26O	flowers	Gan et al., 2013 [23]
7	(5R,10R)-10-Methyl-6-methylene-2-(propan-2-ylidene)spiro[4.5]dec-7-ene	C15H22	flowers	Zeng et al., 2023 [8]
8	α-Muurolene	C15H24	flowers	Zeng et al., 2023 [8]
9	1,3-Cyclohexadiene, 5-(1,5-dimethyl-4-hexenyl)-2-methyl-, [S-(R*,S*)]-	C15H24	flowers	Zeng et al., 2023 [8]
10	(1S,2E,6E,10R)-3,7,11,11-Tetramethylbicyclo[8.1.0]undeca-2,6-diene	C15H24	flowers	Zeng et al., 2023 [8]
11	(2R,3R,6S)-6-Isopropyl-3-methyl-2-(prop-1-en-2-yl)-3-vinylcyclohexanone	C15H24O	flowers	Zeng et al., 2023 [8]
12	(1S,5S,6R)-6-Methyl-2-methylene-6-(4-methylpent-3-en-1-yl)bicyclo[3.1.1]heptane	C15H24	flowers	Zeng et al., 2023 [8]
13	decahydro-4a-methyl-1-methylene-7-(1-methylethenyl)-, [4aR-(4a.α.,7.α.,8a.*β*.)]-	C15H24	flowers	Zeng et al., 2023 [8]
14	Eudesma-2,4,11-triene	C15H22	flowers	Zeng et al., 2023 [8]
Diterpenoid				
1	Neophytadiene	C20H38	flowers	Gan et al., 2013 [23]
Triterpenoids				
1	squalene	C30H50O	flowers	Gan et al., 2013 [23]
2	sasanquasaponin	C58H92O26	seed	Huang et al., 2007 [24]; Chen et al., 2013 [25]
3	yuchasaponin A	C64H99O28	flower	Sugimoto et al., 2009 [26]
4	yuchasaponin B	C64H99O27	flower	Sugimoto et al., 2009 [26]
5	yuchasaponin C	C64H99O28	flower	Sugimoto et al., 2009 [26]
6	yuchasaponin D	C64H99O28	flower	Sugimoto et al., 2009 [26]
7	jegosaponin B	C61H96O27	flower	Sugimoto et al., 2009 [26]
8	camelliasaponin B1	C58H89O26	Seed pomace and root	Kuo et al., 2010 [27]; Zong et al., 2016 [28]; Ren et al., 2019 [29]
9	camelliasaponin B2	C58H89O26	Stem and seed	Yoshikawa et al., 1996 [30]
10	gordonsaponin H	C58H92O26	stem	Fu et al., 2018 [31]
11	theasaponin A1	C57H90O26	seed	Chen et al., 2023 [32]
12	theasaponin A2	C59H92O27	seed	Chen et al., 2023 [32]
13	theasaponin A3	C61H94O28	seed	Zhang et al., 2014 [33]
14	theasaponin A4	C58H92O27	seed	Zhang et al., 2014 [33]
15	theasaponin A5	C60H94O28	seed	Zhang et al., 2014 [33]
16	theasaponin C1	C57H90O25	seed	Zhang et al., 2014 [33]
17	theasaponin E1	C59H90O27	seed	Zhang et al., 2014 [33]
18	theasaponin E2	C59H90O27	seed	Zhang et al., 2014 [33]
19	theasaponin E2 methyl ester	C60H92O27	pomace	Chen et al., 2023 [32]
20	theasaponin E8	C59H90O27	seed	Zhang et al., 2014 [33]
21	theasaponin E9	C61H92O28	seed	Zhang et al., 2014 [33]
22	theasaponin F1	C58H90O27	seed	Chen et al., 2023 [32]
23	theasaponin F2	C60H92O28	seed	Chen et al., 2023 [32]
24	theasaponin F3	C60H92O28	seed	Chen et al., 2023 [32]
25	theasaponin G1	C57H88O25	seed	Zhang et al., 2014 [33]
26	theasaponin H1	C58H90O26	seed	Zhang et al., 2014 [33]
27	oleiferasaponin A1	C59H92O26	seed and pomace	Zhang et al., 2021 [34]
28	oleiferasaponin A2	C63H99O28	seed	Di et al., 2018 [35]
29	oleiferasaponin A3	C63H99O28	pomace	Di et al., 2018 [35]
30	oleiferasaponin B1	C58H90O26	seed	Zhou et al., 2014 [36]
31	oleiferasaponin B2	C61H90O24	seed	Zhou et al., 2014 [36]
32	oleiferasaponin C1	C59H92O26	pomace	Zong et al., 2015 [37]
33	oleiferasaponin C2	C60H95O26	pomace	Zong et al., 2015 [37]
34	oleiferasaponin C3	C63H91O26	pomace	Zong et al., 2015 [37]
35	oleiferasaponin C4	C60H94O27	pomace	Zong et al., 2016 [28]
36	oleiferasaponin C5	C54H84O22	pomace	Zong et al., 2016 [28]
37	oleiferasaponin C6	C65H94O28	pomace	Zong et al., 2016 [28]
38	oleiferasaponin D1	C58H91O25	pomace	Fu et al., 2018 [31]
39	oleiferasaponin D2	C58H89O26	pomace	Fu et al., 2018 [31]
40	oleiferasaponin D3	C58H89O26	pomace	Fu et al., 2018 [31]
41	oleiferasaponin D4	C58H91O26	pomace	Fu et al., 2018 [31]
42	oleiferasaponin D5	C59H91O27	pomace	Fu et al., 2018 [31]
43	oleiferoside A	C63H96O29	root	Li et al., 2014 [38]
44	oleiferoside B	C63H98O29	root	Li et al., 2014 [38]
45	oleiferoside C	C58H92O26	root	Li et al., 2014 [38]
46	oleiferoside D	C64H98O29	root	Li et al., 2014 [38]
47	oleiferoside E	C51H80O19	root	Li et al., 2014 [38]
48	oleiferoside F	C54H82O19	root	Li et al., 2014 [38]
49	oleiferoside G	C60H92O23	root	Li et al., 2014 [38]
50	oleiferoside H	C60H94O23	root	Li et al., 2014 [38]
51	oleiferoside I	C51H78O19	root	Li et al., 2015 [39]
52	oleiferoside J	C63H98O28	root	Li et al., 2015 [39]
53	oleiferoside K	C63H100O28	root	Li et al., 2015 [39]
54	oleiferoside L	C63H96O28	root	Li et al., 2015 [39]
55	oleiferoside M	C63H100O27	root	Li et al., 2015 [39]
56	oleiferoside N	C58H88O23	root	Yang et al., 2014 [40]
57	oleiferoside O	C58H90O23	root	Yang et al., 2014 [40]
58	oleiferoside P	C55H82O21	root	Wu et al., 2015 [41]
59	oleiferoside Q	C55H84O21	root	Wu et al., 2015 [41]
60	oleiferoside R	C53H82O19	root	Wu et al., 2015 [41]
61	oleiferoside S	C53H82O20	root	Wu et al., 2015 [41]
62	oleiferoside T	C53H80O19	root	Wu et al., 2015 [41]
63	oleiferoside U	C52H82O23	root	Zhang et al., 2016 [42]
64	oleiferoside V	C55H82O23	root	Zhang et al., 2016 [42]
65	oleiferoside W	C58H92O26	root	Wu et al., 2015 [41]
66	21*β*,22*α*-diangeloyloxy-3*β*,15*α*,16*α*,28-tetrahydroxyolean-12-en-23-al	C40H60O9	root	Wang et al., 2013 [43]
67	21*β*-angeloyloxy-3*β*,15*α*,16*α*,28-tetrahydroxy-22*α*-(2-methylbutanoyloxy)-olean-12-en-23-al	C40H62O9	root	Wang et al., 2013 [43]
68	21*β*-angeloyloxy-3*β*,16*α*,28-trihydroxy-22*α*-(2-methylbut-anoyloxy)-olean-12-en-23-al	C40H62O8	root	Wang et al., 2013 [43]
69	3-O-*β*-D-xylopyranosyl-(1→2)-*α*-L-arabinopyranosyl-(1→3)-[*β*-D-glucuronopyranosyl-(1→2)]-*β*-D- glucuronopyranosyl-21*β*-acetyl-22*α*-angeloyloxyolean-12-ene-16*α*,28-diol	C57H90O25	stem	Yan et al., 2016 [44]
70	3-O-*β*-D-xylopyranosyl-(1→2)-*α*-L-arabinopyranosyl-(1→3)-[*β*-D-glucuronopyranosyl-(1→2)]-*β*-D- glucuronopyranosyl-22*α*-angeloyloxyolean-12-ene-15*α*,16*α*,28-triol	C59H92O26	stem	Yan et al., 2016 [44]
71	3-O-*β*-D-glucopyranosyl-(1→2)-*β*-D-xylopyranosyl-(1→3)-[*β*-D-galactopyranosyl-(1→2)]-*β*-D- glucuronopyranosyl-22*α*-angeloylox yolean-12-ene-15*α*,16*α*,28-triol	C58H92O25	Stem and pomace	Fu et al., 2018 [31]
72	15*α*,16*α*-Ac-21*β*,22*α*-Ang-23,28-OH-olean-12-ene-3*β*-O-*α*-L-Ara-(1→3)-*β*-D-glucopyranoside	C55H82O21	root	Zhang et al., 2021 [34]
73	15*α*,16*α*-Ac-21*β*-Ang-22*α*-O-(2-methyl-butyryl)-23,28-OH-olean-12-ene-3*β*-O-*α*-L-Ara-(1→3)-*β*-D- glucopyranoside	C55H82O21	root	Zhang et al., 2021 [34]
74	16*α*-Ac-21*β*-Ang-22*α*-O-(2-methyl-butyryl)-23,28-OH-olean-12-ene-3*β*-O-*α*-L-Ara-(1→3)-*β*-D- glucopyranoside	C55H82O19	root	Zhang et al., 2021 [34]
75	16*α*-Ac-21*β*-Ang-22*α*-O-(2-methyl-butyryl)-15*α*,23,28-OH-olean-12-ene-3*β*-O-*α*-L-Ara-(1→3)-*β*-D- glucopyranoside	C53H82O20	root	Zhang et al., 2021 [34]
76	16*α*-Ac-21*β*,22*α*-Ang-23,28-OH-olean-12-ene-3*β*-O-*α*-L-Ara-(1→3)-*β*- D-glucopyranoside	C58H90O23	root	Yang et al., 2015 [45]

## Data Availability

All relevant data cited and analyzed in this review are derived from publicly available published articles, which can be retrieved through academic databases including Web of Science, PubMed, CNKI, and ScienceDirect. No new experimental datasets were generated in this study.

## Abbreviations

The following abbreviations are used in this manuscript:

AACT	Acetoacetyl-CoA thiolase
HMGS	3-hydroxy-3-methylglutaryl-CoA synthase
HMGR	3-hydroxy-3-methylglutaryl-CoA reductase
MK	Mevalonate kinase
PMK	Phosphomevalonate kinase
MVD	Mevalonate diphosphate decarboxylase
DXP	1-deoxy-D-xylulose-5-phosphate
DXS	DXP synthetase
DXR	DXP reductoisomerase
MCT	2-C-methyl-D-erythritol 4-phosphate cytidylyltransferase
CMK	4-diphosphocytidyl-2-C-methyl-D-erythritol kinase
MDS	2-C-methyl-D-erythritol 2,4-cyclodiphosphate synthase
HDS	(E)-4-hydroxy-3-methylbut-2-enyl diphosphate synthase
HDR	(E)-4-hydroxy-3-methylbut-2-enyl diphosphate reductase
GPPS	Geranyl diphosphate synthase
FPPS	Farnesyl diphosphate synthase
GGPPS	Geranylgeranyl diphosphate synthase
mTPS	Monoterpene synthase
sTPS	Sesquiterpene synthase
SQS	Squalene synthase
SQE	Squalene epoxidase
OSC	oxidosqualene cyclase
LUS	lupeol synthase
AS	amyrin synthase
CAS	Cycloartenol synthase
CYP450s	Cytochrome P450 monooxygenases
UGTs	UDP-dependent glycosyltransferases
ACT	Acetyltransferase
16DOX	16α-hydroxylase/16-dioxygenase
GAME	GLYCOALKALOID METABOLISM
SSR	sterol side chain reductase
AKP	Alkaline Phosphatase
MDH	Malate Dehydrogenase
srtA	Sortase A
fbsC	Fibrinogen-binding protein C
neuA	N-acetylneuraminate lyase
cpsE	Capsular polysaccharide synthesis protein E
DPPH	1,1-Diphenyl-2-picrylhydrazyl
NBT	Nitro Blue Tetrazolium
TS	Transition System(Metal Ion Transport System)
CAT	Catalase
T-OAC	Total Oxidative Active Compounds
BHT	Butylated Hydroxytoluene
·OH	Hydroxyl radical
DNCB	2,4-Dinitrochlorobenzene
NF-κB	Nuclear Factor kappa-light-chain-enhancer of activated B cells
IKK	IκB Kinase
NO	Nitric Oxide
C3	Complement Component 3
HMGB1	High Mobility Group Box 1
TSLP	Thymic Stromal Lymphopoietin
IL	Interleukin
OVA	Ovalbumin
TNF-α	Tumor Necrosis Factor-alpha
MAPK	Mitogen-Activated Protein Kinase
PI3K	Phosphatidylinositol 3-Kinase
Akt	Protein Kinase B
mTOR	Mammalian Target of Rapamycin
Bax	Bcl-2-associated X protein
Bcl-2	B-cell lymphoma-2
TG	Triglyceride
TC	Total Cholesterol
HDL-C	High-Density Lipoprotein Cholesterol
AI	Atherogenic Index
SOD	Superoxide Dismutase
MDA	Malondialdehyde
SREBP	Sterol Regulatory Element-Binding Protein
FAS	Fatty Acid Synthase
ACOX	Acyl-CoA Oxidase
CPT	Carnitine Palmitoyltransferase
CTX	Cyclophosphamide
CD68	Cluster of Differentiation 68
ADH	Alcohol Dehydrogenase
sIgA	Secretory Immunoglobulin A
V/C	Villi/Crypt ratio
